# The impact of Health Transformation Plan on natural vaginal delivery and cesarean section frequency in Iran: an interrupted time series analysis

**DOI:** 10.1186/s13104-021-05677-7

**Published:** 2021-07-03

**Authors:** Farhad Lotfi, Saeed Lohivash, Zahra Kavosi, Sakine Owjimehr, Mohsen Bayati

**Affiliations:** 1 School of Health Management and Information Sciences, Health Human Resources Research Center, Shiraz University of Medical Sciences, Almas Building, Alley 29, Qasrodasht Ave, P.O. code: 71336-54361 Shiraz, Iran; 2 Department of Health Economics, School of Health Management and Information Sciences, Shiraz University of Medical Sciences, Shiraz, Iran; 3 Department of Economics, Shiraz University, Shiraz, Iran

**Keywords:** Health care reform, Obstetric delivery, Natural vaginal delivery, Cesarean section, Interrupted time series analysis

## Abstract

**Objective:**

This study was conducted to evaluate the effect of the Iran’s Health Transformation Plan on the frequency of natural vaginal deliveries (NVDs), cesarean sections (CSs), and total deliveries in the Fars province of Iran.

**Results:**

Average number of total deliveries before and after the reform were 3946 and 3810, respectively (p  =  0.164). The ratio of CS to total deliveries in the first study month was 54%. This rate reached 47% in the last month (p  <  0.01). However, it had much fluctuation trend. The ITSA results showed that in the short-run, the NVD rate increased (β  =  492.79, p  <  0.01), the rate of CS decreased (β  =  − 407.09, p  <  0.01), and total deliveries increased (β  =  85.75, p  <  0.724). However, in the long-run, the NVD (β  =  5.74, p  <  0.423), CS (β  =  10.21, p  <  0.189), and total deliveries (β  =  15.96, p  <  0.256) had no significant changes after the reform. Encouraging the NVD package was influential in the short-run but not in the longrun in Iran. Pricing and supply-side policies could not reduce the rate of non-clinical CS on their own. Therefore, paying attention to demand-side policies and changes in consumer behaviors, such as educating the women at the age of pregnancy about the advantages and disadvantages of CS and NVD and correcting misconceptions, could be helpful.

## Introduction

Providing, maintaining, and promoting the health of mothers and infants as two vulnerable groups of society is of particular importance [1]. The prevention of maternal and neonatal mortality and the provision of proper conditions for safe delivery is one of the critical duties of the health system in any country [2].

One of the most important factors affecting maternal and neonatal health is the delivery method. NVD and CS are the two main methods of delivery. Natural delivery itself consist of different manners such as vaginal delivery, natural water birth, labor on squatting position, Hypnobirthing and so on. Natural vaginal delivery (NVD) is the preferred method. Despite the great pain it imposes on the mother, several advantages include the baby’s health and higher intelligence level in adulthood [3].

The other method of delivery is cesarean section (CS), which is an interventional procedure. According to the rules, it is limited to cases in which NVD is impossible or has some risks for the mother or baby [4].

The mortality rate of CS is 4–5 times as much as NVD[5]. The other major complications of CS include endomyometritis and bleeding, urinary tract infection, postpartum infections, and neonatal respiratory distress [5]. Due to elective CS, the maternal mortality rate is 2–3 times more than NVD [6]. The number of disability-adjusted life years (DALY) is 20.6 for CS and 8.8 for NVD per 1000 births [7].

The rate of CS in the world is on the rise [7]. The World Health Organization stated that the rate of CS should not be higher than 10–15% anywhere in the world. Still, according to its report in 2010, in which the statistics from 137 countries were provided, 69 countries had CS rates of over 15%, and only 14 countries had standard CS rates [8]. The highest and lowest percentages of this type of delivery were in Brazil (45.9%) and the African country of Chad (4%) [9]. In Iran, the rate of CS has increased significantly during the past 2 decades and has changed from 35% in 2000 to 47.9% in 2009 [10]. According to studies, the mean rate of CS in some private hospitals was even close to 90% in recent years. More precisely, an average of 48% of deliveries in Iran are through CS [11].

The high rate of CS in Iran and its growing trend in recent years, on the one hand, and the change in demographic policies on the other hand, require special measures to be taken to control the high rate of this kind of delivery [12]. If the rate of CS continues to increase, it will impose significant costs to the health system and community and cause maternal and neonatal health problems [13–15].

Regarding what was mentioned above, the Ministry of Health (MoH) developed a package in a reform called Health Transformation Plan (HTP) to promote NVD and reduce CS. The plan has been implemented in all hospitals affiliated with the MoH since April 2014. It has been designed to motivate to increase the NVD rate and reduce the rate of CS through the use of a combination of strategies. The overall goal of the HTP was to promote maternal and neonatal health indicators by lowering the rate of CS. Its objectives included reducing CS by 10% by the end of 2014, increasing pregnant women’s satisfaction, reducing out-of-pocket payments to zero for natural deliveries in public health centers, optimizing the physical environment of maternity wards, and increasing the incentives for service providers to promote NVD [14].

So far, no comprehensive study has been done on the success of the HTP in reducing CS and increasing NVD. Moreover, few studies were limited to descriptive surveys or investigating the mean differences before and after the plan’s development. Therefore, the present study was designed to analyze the short-term and long-term effects of the HTP on the rates of NVD and CS.

## Main text

### Methods

The statistical population in this study included all the hospitals in Fars province affiliated to Shiraz University of Medical Sciences. The data were collected by referring to the Statistics Administration of Shiraz University of Medical Sciences. The required data were extracted monthly from 2 years before to almost 2 years after the development of the HTP. The study variables included the total number of deliveries, the number of NVDs and CSs performed per month in all the intended hospitals.

The Interrupted Time Series (ITS) analysis was used to investigate the effect of the HTP on NVD and CS rates. Using this method, we studied the effect of the HTP on the indicators’ level and trend.

The ITS model is one of the most advanced and applied statistical methods used to determine the impact of interventions and evaluate different health sector policies. In this model, the two variables used to indicate the effect of a policy or intervention are the level and trend, which determine the immediate and long-term impacts of the interventions, respectively. The following segmented regression model was estimated for the present study:

$${Y}_{t}={\beta }_{0}+{\beta }_{1}{T}_{t}+{\beta }_{2}{X}_{t}+{\beta }_{3}{T}_{t}{X}_{t}+{e}_{t}$$

*Y*_*t*_ represents the dependent variable (the total number of deliveries per month), and *T* represents time. *X*_*t*_ shows the intervention, which is the package to motivate NVD. *T*_*t*_*X*_*t*_ describes the interactive effects of time and intervention, and *e*_*t*_ shows the error term.

The *X*_*t*_, which was a dummy variable, was given the value of 1 after the intervention (May 2014), and 0 was given before that. In the present study, the total time points was 60 months (3/2012–2/2017), 26 months of which were before the intervention (0) and 34 months were after the intervention (1).

In this model, β_0_ represents the intercept or the variable (total deliveries/NVDs/CSs) level before the intervention, β_1_ represents the time trend before the HTP, β_2_ shows the immediate impact of the HTP, and β_3_ indicates the long-term impact of the HTP.

To prevent spurious regression estimation, the stationary of the variables was initially evaluated using the Dickey Fuller unit root test. It was found that the null hypothesis on the existence of the unit root for all indicators was rejected (p  <  0.05), and the time series was stationary for all variables.

### Results

As shown in Table 1, the CS rate in the current study was about 50% during the study years, regardless of the deliveries in private centers. The rate of CS was 52% at the beginning and 50% at the end of the study’s period. It had a falling trend after implementing the HTP, but it was still much higher than the standard rate. Although there was some fluctuation after the implementation of the HTP, it eventually reached 50%. The results showed no significant difference between the mean number of NVDs and CSs before and after the HTP. On average, the number of CSs decreased by 338 per month after implementing the plan, and about 202 more NVDs per month were reported after the implementation of the plan. The total number of deliveries before the plan was about 3946, which reached 3810 after implementing the HTP.

**Table 1:** Monthly average of total delivery, NVD, CS, and rate of CS in population before and after HTP

Variables	Mean (standard deviation)			t-statistic (p value)
	Mean	Mean before HTP	Mean after HTP	
NVD	1930.81 (231.99)	1816.30 (226.19)	2018.38 (198.06)	− 3.68 (0.000)
CS	1938.56 (268.54)	2130.38 (229.02)	1791.88 (195.04)	6.17 (0.000)
Total delivery	3869.38 (375.38)	3946.69 (436.56)	3810.26 (315.03)	1.40 (0.164)
Cs/total	50.06 (4.40)	54.03 (1.77)	47.02 (3.23)	9.95 (0.000)

Table 2 shows that before implementing the HTP, the initial level of NVD was positive, and its trend was falling and significant (p  <  0.037). Once the plan was implemented, the NVD rate increased during the plan instantaneously (p  <  0.000). The NVD trend declined after the plan but not significantly (p  =  0.067), and the decline had a milder slope than the pre-plan period.

**Table 2:** Estimates of segmented regression for Effects of HTP on NVD and CS rate in Iran

		NVD	CS	Total delivery
Pre HTP	Initial level	1976.96 (0.000)	2172.04 (0.000)	4149.01 (0.000)
	Initial trend	− 12.85 (0.037)	− 3.33 (0.646)	− 16.18 (0.216)
Post HTP	Trend after reform	− 7.10 (0.067)	6.88 (0.011)	− 0.22 (0.965)
	Change in level after reform	492.79 (0.000)	− 407.09 (0.003)	85.70 (0.724)
	Change in trend after reform	5.74 (0.423)	10.21 (0.189)	15.96 (0.256)
Overall model significance	F-statistic	8.02 (0.000)	17.32 (0.000)	1.87 (0.144)

The initial level of CS was positive and significant before the HTP (p  <  0.000), and its trend was falling significantly (p  =  0.646). In other words, before the plan, the initial level of CS was about 2172 per month, however the trend of CS was insignificantly decreasing with slope − 3.3. However, during the implementation of the plan, the CS rate had an immediate reduction (p  <  0.003), and after the plan, an incremental rate was obtained, which was not significant (p  =  0.189).

The initial level of the total deliveries was positive and significant before and after the implementation of the plan (p  <  0.000).Its trend also was declining before the plan (p  =  0.216). During the implementation of the HTP, there was an immediate increase (p  =  0.724), but after the plan, the total delivery trend was decreasing insignificantly (p  =  0.965).

**Fig. 1:**
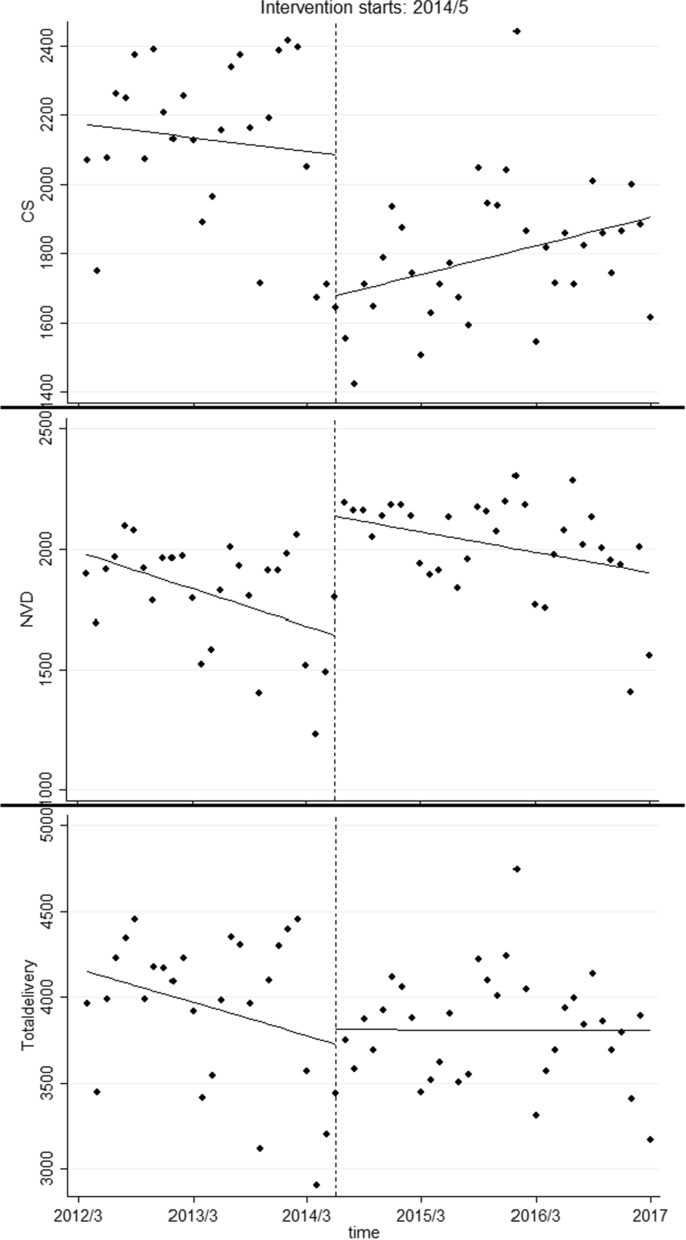
Fitted segmented regression lines for CS, NVD and total delivery before and after the HTP

The findings also have been presented in Fig. 1.

The F statistic in the model represents the overall model’s significance.

### Discussion

The present study aimed to investigate the success of the HTP in encouraging NVDs and reducing CSs rates.

The study results showed that in the short run, the NVD rate increased, and the rate of CS decreased. Hence, the policy was influential in the short term.

Few studies conducted in Iran confirm this finding. For example, Piroozi et al. [1] showed in their descriptive study that the rate of CS in Sanandaj University hospitals had decreased by 14% in the first year of implementing the plan. In addition, Moradi et al. indicated that an effective factor in the selection of NVD by pregnant mothers in the short term was that such a kind of delivery was free [15, 16].

The decline in the NVD rate became lower after implementing the plan and continued at a slower pace. Besides, the reducing trend of CS decreased after the HTP. Therefore, the policy was not effective in the long run.

The unsuccessfulness of the reform for the promotion of NVD in the long term can have several multidimensional reasons. Some of which are clinical factors that make CS inevitable, such as high risk of NVD for mothers [17], high maternal age at first delivery [18], and undergoing CS as the first delivery. According to evidence, a significant percentage of CSs in Iran cannot be medically justified [3]. Other non-clinical factors, such as cultural factors, lack of awareness of the women about the complications of CS, socioeconomic factors, and poor implementation of the policy over time, can be considered the most important factors in failure the plan in the long run.

Arikan et al. [19] suggested that misconceptions and wrong cultural factors, such as undergoing no pain during the operation, no risk of injury to the fetus, prevention of rupture and deformation of the genitalia, and fear of NVD, were the reasons for Iranian women to choose CS. In a study carried out in Turkey, two-third of pregnant women preferred to give birth through elective CS, and about 50% of them believed that choosing the type of delivery was the right of any woman [20]. In a study in Australia, different clinical decisions and elective CS were considered the factors that were increasing the number of CSs among pregnant women [21]. Another study in Brazil showed that the pregnant mothers’ fear of labor pain and various conditions of NVD were the reasons for their tendency toward CS.

The lack of knowledge about the complications of CS and the benefits of NVD to the mothers and babies are considered one of the critical factors. Mousavi et al. concluded that increasing the awareness of pregnant mothers about natural delivery and their understanding of the importance of this kind of delivery could be effective in choosing the delivery method [22].

Socioeconomic factors might also influence the selection of CS as a delivery method. Béhague et al. [23] stated that social and economic inequalities led more pregnant mothers to CS. In Turkey, the increased CS rate by 51.9% in 2013 was attributed to the reasons such as CS insurance coverage, living in more prosperous areas, higher economic status, and being first-pregnant [24].

Anticipating vaginal birth as a painful and lengthy process, low cultural acceptance and tariffs of different delivery procedures, were most important factors in choosing delivery methods from Iranian obstetricians points of view [25].

One of the other factors was poor implementation and financing of the natural delivery package and whole HTP. For example, the delays in paying the staff and doctors could reduce their incentive to properly implement the plan. Piroozi et al. [1] stated that financial encouragements and increased tariffs for physicians and midwives at the beginning of the plan were effective factors in encouraging them to reinforce the incentive for pregnant women to opt for NVD. Still, later, reduced resources and deferral of the payments of physicians and maternity personnel made them less likely to continue the plan properly.

In addition, one of the reasons for the increased rate of CS in public hospitals could be the referral of high-risk mothers to government delivery centers for undergoing CS.

According to the findings of this study, although the rate of CS, in the long run, was increasing more rapidly compared to the time before the implementation of the plan, its quantitative number was still lower than the latest data before the plan was implemented.

### Conclusion

Encouraging NVD package was effective in the short-run but not in the long-run in Iran. This study indicated that pricing policies and supply sector policies could not reduce the rate of non-clinical CS on their own. Therefore, paying attention to demand sector policies and fundamental changes in consumer behaviors, such as educating the women at the age of pregnancy about the advantages and disadvantages of CS and NVD and correcting misconceptions, could be helpful.

## Limitations

One of the main limitations of the present study was the non-access to private-sector data. However, given that the rate of CS in private hospitals was much higher than in the public ones, the results could change if the deliveries in the private sector were considered.

## Data Availability

The datasets generated and/or analyzed during the current study are not publicly available due to data sharing policies of the vice chancellery for research affairs of Shiraz University of Medical Sciences but are available from the corresponding author on reasonable request.

## Abbreviations

CS: Cesarean section
HTP: Health Transformation Plan
ITS: Interrupted time series
MoH: Ministry of Health
NVD: Natural vaginal delivery
